# EStreams: An integrated dataset and catalogue of streamflow, hydro-climatic and landscape variables for Europe

**DOI:** 10.1038/s41597-024-03706-1

**Published:** 2024-08-13

**Authors:** Thiago V. M. do Nascimento, Julia Rudlang, Marvin Höge, Ruud van der Ent, Máté Chappon, Jan Seibert, Markus Hrachowitz, Fabrizio Fenicia

**Affiliations:** 1 Eawag: Swiss Federal Institute of Aquatic Science and Technology, Dübendorf, Switzerland; 2 Department of Geography, University of Zurich, Zurich, Switzerland; 3 Department of Water Management, Faculty of Civil Engineering and Geosciences, Delft University of Technology, Delft, Netherlands; 4 Széchenyi István University, Department of Transport Infrastructure and Water Resources Engineering, Győr, Hungary

**Keywords:** Hydrology, Environmental sciences

## Abstract

Large-sample hydrology datasets have become increasingly available, contributing to significant scientific advances. However, in Europe, only a few such datasets have been published, capturing only a fraction of the wealth of information from national data providers in terms of available spatial density and temporal extent. We present “EStreams”, an extensive dataset of hydro-climatic variables and landscape descriptors and a catalogue of openly available stream records for 17,130 European catchments. Spanning up to 120 years, the dataset includes streamflow indices, catchment-aggregated hydro-climatic signatures and landscape attributes (topography, soils, geology, vegetation and landcover). The catalogue provides detailed descriptions that allow users to directly access streamflow data sources, overcoming challenges related to data redistribution policies, language barriers and varied data portal structures. EStreams also provides Python scripts for data retrieval, aggregation and processing, making it dynamic in contrast to static datasets. This approach enables users to update their data as new records become available. Our goal is to extend current large-sample datasets and further integrate hydro-climatic and landscape data across Europe.

## Background & Summary

Large-sample datasets of hydrological variables across many catchments and long time periods are crucial for understanding and predicting hydrological variability in time and space^1,2^. These datasets are increasingly in demand due to the rise of data-intensive machine learning models^3^.

Following the publication of the MOPEX dataset in the early 2000s, there has recently been a broad movement to making large-sample hydrology (LSH) datasets available. Many of those were developed inspired by the Catchment Attributes and MEteorology for Large-sample Studies (CAMELS) initiative that compiled and made available full datasets for the contiguous United States^1^. Many countries and regions have embraced these or similar initiatives, including Australia^4^, Brazil^5^, Chile^6^, Great Britain^2^, Switzerland^7^, Central-Europe^8^, North America^9^, China^10^, Central Asia^11^ and Iceland^12^.

At the global scale, there are already some collection efforts for hydro-meteorological data. The Global Streamflow Indices and Metadata Archive (GSIM)^13,14^ provides streamflow indices for 35,000+ locations around the globe, but no extensive set of catchment landscape and meteorological attributes. Recently another global streamflow indices time series initiative took place enlarging the analysis to 41,000+ river branches worldwide and using different streamflow signatures to enrich the flow regime analysis^15^. Considering streamflow records, the Global Runoff Data Centre (GRDC)^16^ provides data for 10,000+ stations, but similar to the previous datasets, no catchment attributes and meteorological forcing time series are available. In addition, the GRDC data is only updated episodically, while the others do, to our knowledge, not provide any updates. More recently the Caravan^3^ dataset compilation was published as a global initiative for standardizing already open-source published streamflow datasets of initially 6,830 catchments, where catchment attributes and meteorological forcing were derived from gridded global products.

While global datasets offer easy access, they come with limitations. Firstly, their spatial coverage remains restricted, offering only a fraction of data available from national providers worldwide. The Caravan dataset, for example, originally covered Europe for only Great Britain, Austria and the Danube catchment as far downstream as the city of Bratislava (Slovakia). By now, there are multiple extensions for Denmark, Israel, Switzerland, Spain, Iceland and, most recently, a GRDC extension^17^ adding another 25 countries globally. Yet, for eastern and southern Europe publicly available data is still difficult to access. Secondly, such datasets are also limited in their temporal extent. For example, the CAMELS-GB^2^ covers the period from 1970 to 2015, while the LamaH-CE dataset^8^ spans from 1981 to 2017. Thirdly, existing large sample hydrology datasets, including the CAMELS databases, lack extensibility, making the accommodation of newly available data challenging.

Although most countries collect daily streamflow data at numerous river gauging stations, compiling a comprehensive hydrological dataset from this information presents significant challenges. Firstly, access to these data can be challenging. Some countries offer this data on the official websites of government agencies or associated data providers, while others provide it upon request. Official government websites are frequently available only in national languages, adding an extra layer of complexity. Gaining access can be intricate, involving navigation to a selection of stations and periods, which need to be downloaded individually. Secondly, substantial formatting and pre-processing are often necessary before the data can be effectively utilized. Finally, redistribution restrictions may hinder the republishing of country-specific data. These obstacles pose significant barriers to hydrological analyses of catchments in large-sample investigations, particularly given the short timeframes of typical research projects.

Here, we present “EStreams”, a platform consisting of two distinct products: (1) an extensive streamflow catalogue together with Python scripts for data direct access at the individual data providers and (2) a dataset of weekly, monthly, seasonal and annual indices, of streamflow, together with the associated catchment-averaged hydro-climatic signatures, meteorological time series and landscape descriptors for 17,130 catchments across 41 countries over pan-European territory. Currently, the dataset covers the period of 1900–2022.

While the focus of EStreams is on streamflow, the EStreams dataset also contains catchment aggregated meteorological forcing and landscape descriptors, typically necessary for hydrological analyses. These indices and descriptors were derived from various open source datasets and include climate^18^, geology^19,20^, hydrology and topography^21–24^, land use and land cover^25–27^, soil types^28–30^ and vegetation characteristics^31,32^. Similarly to streamflow, national providers often have more accurate information for such auxiliary data, but seldom they are easily accessible.

Unlike existing global datasets, which are relatively “static” as not easily updatable with new stations or recent time periods, EStreams is designed as “dynamic” by linking users to the original data providers. While “static” datasets may offer more accurate quality checks and are well-suited for applications such as benchmarking methods and models, many practical applications benefit from using the most up-to-date and dense data. This is particularly true for tasks like accurate streamflow predictions using data-intensive machine learning models.

Hence, our main contributions with this work are:

i. Introducing the currently most extensive and extensible integrated collection of weekly, monthly, seasonal and annual indices of streamflow for Europe, along with catchment-aggregated meteorological and landscape variables (dataset).
ii. Providing detailed metadata for streamflow gauges, including catchment boundaries, and a catalogue of the corresponding data providers.
iii. Allowing reproducibility and extension by making available all codes used to retrieve the source data and aggregate them by catchment in an easy-to-use workflow, allowing users to directly and readily access the desired data from data providers.

**Fig. 1:**
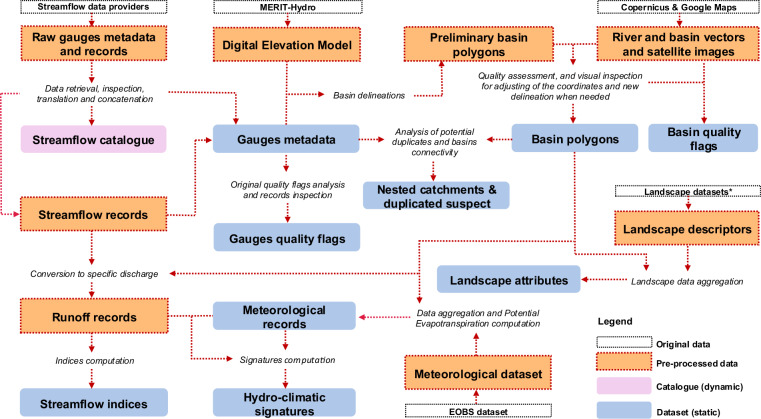
Framework of the methodology adopted in EStreams for deriving the Streamflow Catalogue, and the Dataset. The boxes with dashed lines represent the original, and the intermediate (pre-processed) data used in EStreams. The outputs are shown in pink (catalogue) and blue (dataset). *The landscape datasets encompass topography, soils, geology, hydrology, vegetation and land cover.

The methodology employed to process the source data and obtain the current dataset and catalogue is illustrated in Fig. 1. This figure highlights the primary data sources, the general procedure, and the final outputs of EStreams. A detailed description of each step is provided in the Methods sections.

## Methods

### Streamflow data

#### Available stations

**Fig. 2:**
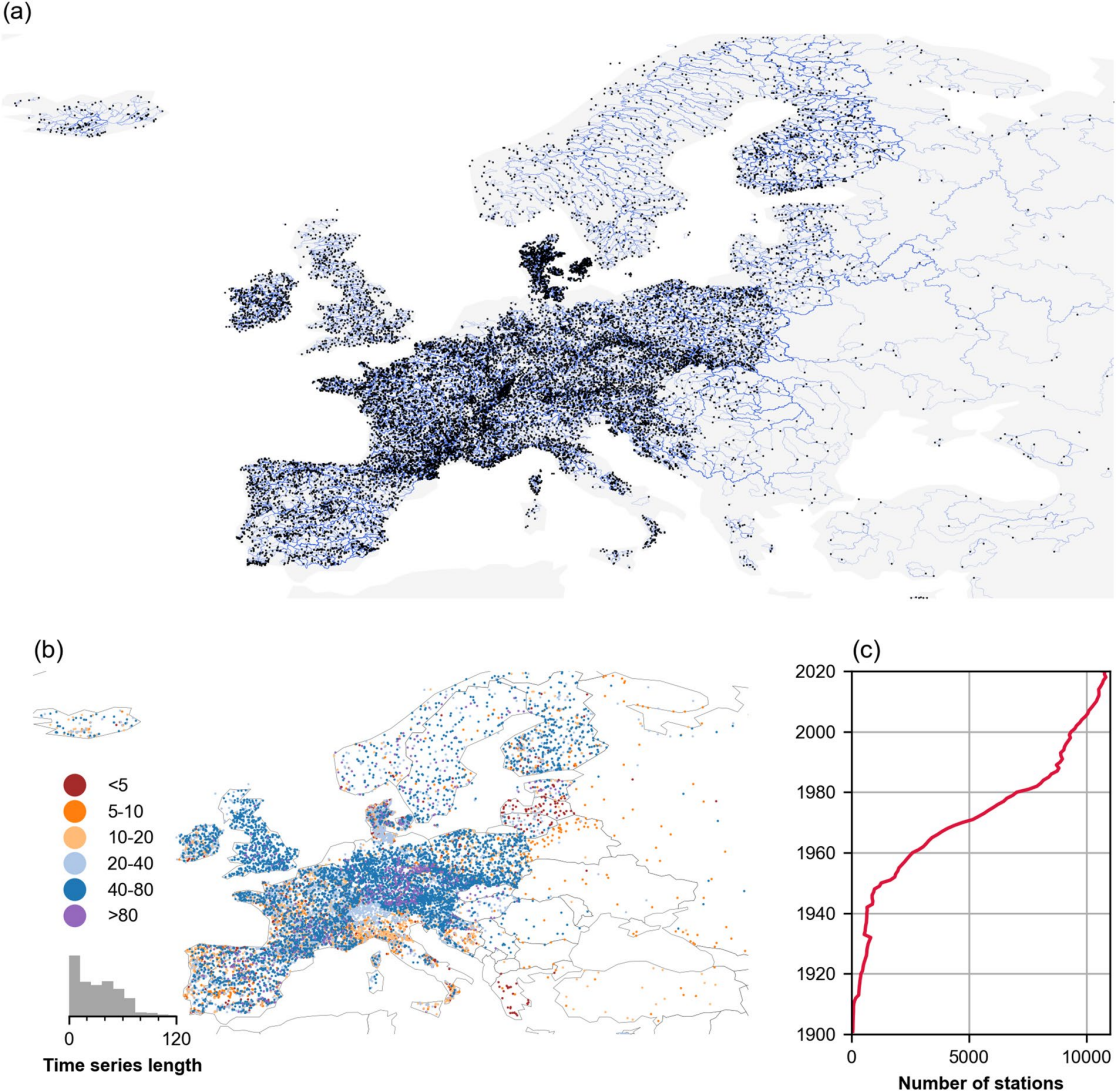
(**a**) Spatial distribution of the 17,130 streamflow gauges currently included in EStreams (in black dots) with their catchment boundaries in background (in blue) over Europe. (**b**) Spatial distribution of the streamflow with the colors representing the time series length in years. (**c**) Temporal evolution of station coverage. The plot shows the number of active stations in a given year, Although the curve accounts for dismissed stations, it still shows an increasing trend. Basemap from GeoPandas^104^.

Daily streamflow data from 17,130 European river catchments with varying sizes and characteristics were aggregated from 41 countries and more than 50 different data providers. In some countries, such as Italy and Germany, multiple data providers contributed to the dataset. Figure 2a shows the distribution of the gauges with their respective catchment boundaries in the background. As can be seen in the figure, there is a significant variability in terms of station density, which is the highest in central Europe and the lowest in the South and the East. The time series records span the period 1900–2022, with varying length for each catchment, as shown in Fig. 2b. Central Europe features the longest time series, with many stations with records extending over 80 years. Figure 2c shows the evolution of the number of stations with measurements at a given time accounting for the discontinuity of stations over time. The plot shows an increasing trend in the number of gauging stations with concurrent records.

The streamflow records were selected based on the following criteria: (i) they were available from official authorities in their respective country or from a recent open-access dataset, and (ii) they were open-source and easily accessible either via the internet or by e-mail request. The latter point emphasizes that no dataset requiring purchase for non-commercial access were included. It is important to note that freely available data do not necessarily come with a free redistribution license. Therefore, we cannot and do not make raw daily streamflow data directly available. Should the source data be necessary, we provide the EStreams catalogue of data sources to allow users easy and direct data access from the original repositories, including codes and instructions for data download and formatting. Compared to static databases of pre-compiled datasets currently available, our approach has two main advantages:

i. Users can tailor the download to determine the desired spatial and temporal coverage, also making use of the provided descriptive statistics of the source data, such as regime characteristics or catchment properties.
ii. Users can access the most up-to-date information directly from the data sources.

Table 1 provides an overview of the contributing countries, the number of streamflow gauges, and the data providers. France has the highest number of gauges (4,968), followed by Germany (2,093) and Spain (1,440). In contrast, Bulgaria (8 gauges) Moldova (2) and North Macedonia (1) have the lowest numbers of gauges.

**Table 1:** Overview of streamflow time series data available per country/region, with information about number of stations and data providers.

Country/region	Code	Stations	References
Austria	AT	582	BML^49^
Bosnia and H.	BA	91	GDRC^16^; FHMZBIH^50^
Belgium	BE	230	VW^51^; SPW^52^
Bulgaria	BG	8	GRDC^16^
Belarus	BY	51	GRDC^16^
Switzerland	CH	298	BAFU^7,53^
Cyprus	CY	14	GRDC^16^
Czechia	CZ	566	CHMI^54^
Germany	DE	2,093	LHW^55^; ASOEAG^56^; Umweltportal^57^; ELWAS-WEB^58^; NLWKN^59^; HLNUG^60^; GKD^61^; LUBW^62^; WB^63^; LBAW^64^; MKUEM^65^; LUBN^66^; BFG^67^
Denmark	DK	1,000	ODA^68^
Estonia	EE	67	GRDC^16^
Spain	ES	1,440	CEDEX^69^
Finland	FI	669	FEI^70^
France	FR	4,968	BanqueHydro^71^
Great Britain	GB	671	NRFA^72^
Greece	GR	31	GRDC^16^; OHIN^73^; HCRM^74^
Croatia	HR	317	DHZ^75^
Hungary	HU	98	GRDC^16^; OVF^76^
Ireland	IE	464	EPA^77^; OPW^78^
Iceland	IS	111	LamaH-Ice^12^
Italy	IT	767	GRDC^16^; ISPRA^79^; APC Abruzzo^80^; CFRA Valle d’Aosta^81^; ARPAE Emilia-Romagna^82^; ARPA: Umbria^83^, Sardegna^84^, Lombardia^85,86^, Toscana^87^, Piemonte^88^; ARPAL Liguria^89^; ARPAV Veneto^90^; SPRUD Trentino^91^
Lithuania	LT	76	GRDC^16^
Luxembourg	LU	19	NGGL^92^
Latvia	LV	61	GRDC^16^
Moldova	MD	2	GRDC^16^
Macedonia	MK	1	GRDC^16^
N. Ireland	NI	51	NRFI^72^
Netherlands	NL	17	RWS^93^
Norway	NO	189	NVE^94^
Poland	PL	1,287	IMGW-PIB^95^
Portugal	PT	280	SNIRH^96^
Romania	RO	18	GRDC^16^
Serbia	RS	18	GRDC^16^
Russia	RU	98	GRDC^16^
Sweden	SE	290	SMHI^97^
Slovenia	SI	117	ARSO^98^
Slovakia	SK	21	GRDC^16^
Turkey	TR	28	GRDC^16^
Ukraine	UA	21	GRDC^16^

#### Streamflow gauges labelling

After the collection of the streamflow data and gauge information from each provider, the individual datasets were collated into a single dataset. In this process, each gauge was labelled with a unique 8-digit code. Consequently, each catchment was renamed according to its respective streamflow gauge. The 8-digit codes were generated using the following logic: the first two digits represent the country/region, the next two digits represent specifications about the data provider within regions that had more than one official provider, and the last four digits refer to the gauge counter for each country/region. For example, the gauge GB000045 represents Great Britain (GB), with only one provider (00), and the gauge number 0045. Similarly, ITIS0001 represents Italy (IT), with ISPRA (IS) as the data provider, and gauge number 0001. The gauges with records obtained from GRDC have the second two digits as “GR” (e.g., LVGR0001) to facilitate identification. This standardization ensures that all gauges are consistently labelled, providing users with a clear indication of the source and the number of records.

#### Identification of duplicate gauges

When compiling large streamflow datasets, there is a possibility of having duplicate records within the dataset that need to be identified and removed. This issue can arise when combining information from multiple sources and even within datasets obtained from a single data provider. To identify suspected duplicate records, we used a similar approach as used by the GSIM^13^, where for gauges originating from distinct data providers, we identified potential duplicate gauges by examining similarities in gauge and river names. We employed the Jaro-Winkler distance metric to quantify alphanumeric similarity, as discussed by Christen, 2012^33^ with a threshold set at 0.70. We additionally considered spatial proximity, constraining pairs of stations within 1 km of each other. For gauges originating from the same data provider, we selected stations within a spatial proximity of 50 m and a delineated area difference below 1%. Gauges meeting these criteria were flagged as potential duplicates. The list of potential duplicates for each gauge is contained in the attribute ***duplicated_suspect*** within the gauges’ layer in the final EStreams dataset. Notably, all potential duplicates are preserved in EStreams, giving users the flexibility to choose their preferred station and data provider when duplicates are found. This approach ensures that users can tailor their dataset according to their specific needs and preferences.

#### Quality flags of records

Quality control of streamflow data is essential before undertaking any hydrological study. While some data providers include quality flags with each published record, this practice is not consistently available. Automatic checks are available but may be subjective, and their effectiveness has not yet been fully investigated^34,35^. For example, Do, 2018^13^ employed an automatic detection criterion to identify and filter potentially suspect records based on negative values, consecutive repetitions, and outliers. However, these filtering criteria are not always reliable, as pointed out by Chen, 2023^15^.

In this work, following the approach utilized by Chen, 2023^15^, we adopt a two stages approach for quality checking the data, the first oriented at individual data points, and the second assessing the entire record. The first stage is primarily based on the quality flags from the original providers, when available, which for consistency are reclassified into four categories: “missing”, “no-flags”, “suspect” and “reliable”. First, all negative values were replaced with “not a number” (*NaN)* and flagged as “missing”. Then, values with a quality flag given by the data providers had their original labels reclassified as either “reliable”, “suspect” or “missing”. Finally, all data without a quality flag from the original providers were classified as “no-flag”. A complete overview of the mapping between the original flags and our four flags system is available in Supplementary Table 1.

In the second stage, we assessed the overall reliability of each entire time series based on the fraction of problematic data points as determined in the previous stage. This classification considered five criteria outlined in Table 2.

**Table 2:** Criteria used for the quality assessment of the streamflow gauges as in Chen, 2023^15^.

Quality flag (gauge)	Criterion
A	More than 95% of the gauge records flags are “reliable”
B	More than 95% of the gauge records flags are “reliable” or “no-flag”
C	Less than 10% of the gauge records flags are “missing”
D	Less than 20% of the gauge records flags are “missing”
E	More than 20% of the gauge records flags are “missing”

When one station met multiple criteria simultaneously, the highest-level flag was applied.

**Fig. 3:**
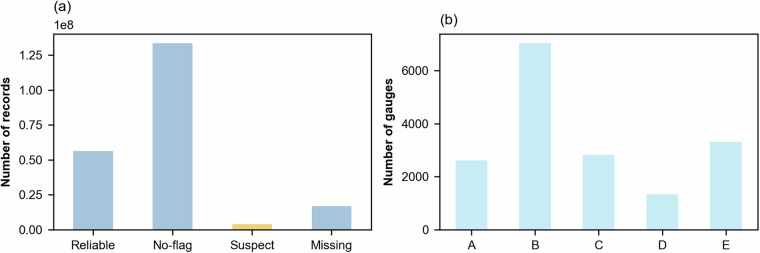
(**a**) Histogram of the streamflow data points according to their four data quality flags and (**b**) Histogram of the number of gauges according to their integrated data quality flag.

A total of 7,430 stations had quality flags from their providers (about 43% of the total). Figure 3a shows that approximately 134 million data points (63.4% of the total) were classified as “no-flag”, 56 million data points (26.7%) as “reliable”, 3.9 million data points (1.9%) as “suspect”, and 16.8 million data points (8%) as “missing”. Regarding the gauge’s quality classification, Fig. 3b shows that most stations were categorized as either Class A or B (9,652), followed by Class E (3,317), Class C (2,827) and Class D (1,334). This classification allows users to filter the data depending on their needs. It is noteworthy that many national providers may offer only high-quality data for download. Therefore, even without explicit quality flags, the data can often be assumed to come from reliable stations. The quality flag for each gauge’s records is stored as the attribute ***gauge_flag*** within the gauges’ layer in the final EStreams dataset.

### Basin delineation

Since catchment boundaries shapefiles were rarely available from national providers, this work adopted a semi-automatic delineation of catchment boundaries corresponding to streamflow gauges using Python scripts and QGIS software. We used the “delineator” python package^36^, which determines catchment boundaries using hybrid vector and raster-based methods. This package requires as input the latitude and longitude coordinates of the streamflow gauges and uses the MERIT-Hydro Digital Elevation Model (DEM)^21^. MERIT-Hydro is a digital elevation model developed to remove multiple error components from the existing spaceborne DEMs (SRTM3 v2.1 and AW3D-30m v1).

To appraise the accuracy of the delineated area, catchments were split into two categories: (i) catchments with a reported area from the data providers and (ii) catchments without this information. For gauges with available official catchment areas, the reported area was compared to the derived area, and the following workflow was adopted:

i. First, we computed the “relative area difference” *A*_rel_ as defined in Eq. 1. If |*A*_rel_| was below 10%, regardless of catchment size, the delineation was accepted, and the catchment was labelled with a quality flag of “0”.
ii. Otherwise, the catchment delineation was visually inspected, potentially corrected as described below, and assigned a specific quality flag as detailed in Table 3, which provides an overview of the flags used and number of gauges corresponding to each flag.

**Table 3:** Description of the catchment area quality flags adopted for the current catchment delineations and overview of the number of catchments per group.

Basin area quality flag	Number of gauges	Description
0	12,801	|*A*_rel_| below 10%.
1	164	|*A*_rel_| below 10% after moving the gauge location.
2	1,037	|*A*_rel_| above 10% or no reported area available, but delineation visually compared to other delineations from down and upstream gauges labelled “0”, Google Maps satellite imagery and to the EU-Copernicus River network.
3	369	|*A*_rel_| above 10% or no reported area available, but delineation visually compared to Google Maps satellite imagery and to the EU-Copernicus River network.
4	343	|*A*_rel_| above 30% or no reported area available, but delineation compared to EU-Copernicus River network.
5	68	|*A*_rel_| above 10% or no reported area available, and delineation manually adjusted using EU-Copernicus in addition to MERIT-Hydro.
6	11	Similar to “5”, but still with |*A*_rel_| above 30% or no reported area available.
888	64	|*A*_rel_| above 10% or no reported area available, but location in areas under high human influence, such as canalization and water exports and in karstic regions.
999	2,273	|*A*_rel_| above 10% or no reported area available, and delineation eventually not accepted after visual inspection.

1 $${{\boldsymbol{A}}}_{{\boldsymbol{rel}}}={\bf{100}}\times \frac{{{\boldsymbol{A}}}_{{\boldsymbol{EStreams}}}-\,{{\boldsymbol{A}}}_{{\boldsymbol{official}}}\,}{{{\boldsymbol{A}}}_{{\boldsymbol{official}}}}$$

where *A*_EStream_ is the calculated area in EStreams and *A*_official_ is the reported official area.

The visual inspection was made using the river networks from both the MERIT-Hydro and EU-Hydro datasets^37^, Google Maps satellite imagery, and nearby catchments delineated and labelled with a quality flag of “0”. These three data sets were used as they represent independent sources and offer a good trade-off for evaluating the catchment delineation usability.

During the visual inspection, it was observed that some boundary discrepancies could be corrected with an adjustment in the streamflow gauge location. We assumed that uncertainties in the georeferenced system or the presence of close-by river branches could cause these discrepancies. For those catchments, the gauge location was moved (snapped) to the closest point within the MERIT-Hydro River network based on the gauge’s river and location names.

Catchments with |*A*_rel_| below 10% after the snap were labelled with a quality flag “1” indicating accepted delineation after the snap. The remaining catchments were classified with the criteria detailed in Table 3.

It is important to note that for some situations where human-influence such as canalization, water exports and specific lithologies like karstic systems, the actual catchment boundary delineation remains challenging. Hence, for catchments where |*A*_rel_| was above 10% and the visual inspection indicated such situations, we assigned a quality flag of “888”.

Finally, catchments where |*A*_rel_| was above 10%, and were not visually adjusted or accepted, were assigned to a quality flag ‘‘999”.

**Fig. 4:**
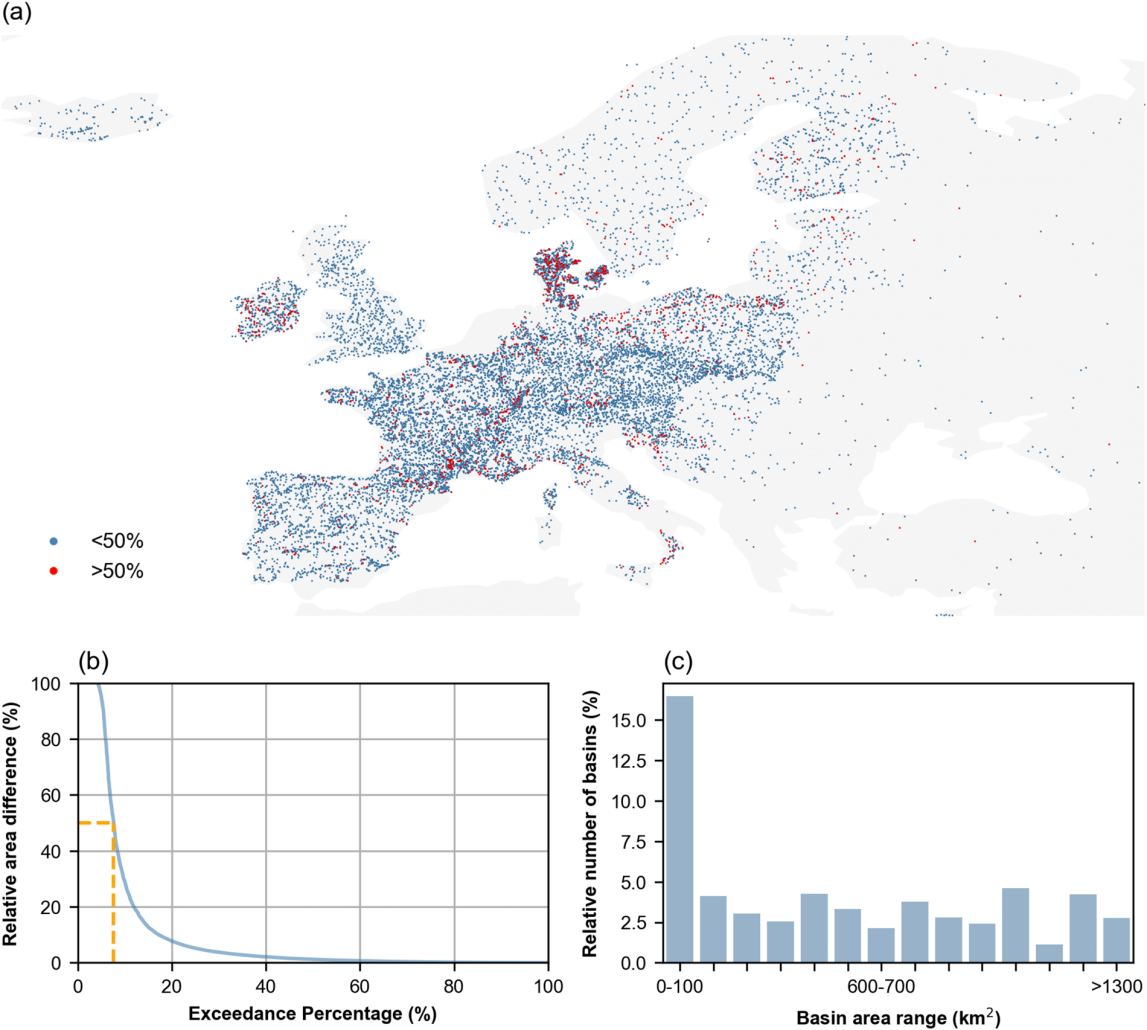
(**a**) Relative absolute area difference |*A*_rel_| above 50% (in red) and below 50% (in blue). (**b**) Exceedance percentage of the |*A*_rel_|; the orange line marks the exceedance percentage corresponding to a |*A*_rel_| of 50%. (**c**) Bar plots showing the relative number of basins with areas above 50% for different basin area ranges (e.g., 0–100 km², 100–200 km², and >1,300 km²) relative to the total number of basins in each range. Basemap from GeoPandas^104^.

Out of a total of 17,130 stations, 15,775 (92%) had a reported catchment area from the data providers. Figure 4a shows the distribution of these streamflow gauges divided into two classes: gauges with |A_rel_| above 50% (in red), and those with |*A*_rel_| below 50% (in blue). Generally, gauges with high area discrepancies are located in regions of low relief, partly canalized landscapes and with high presence of lakes such as in Denmark, Sweden and Croatia.

Figure 4b shows the exceedance percentage of |*A*_rel_| of these 15,775 catchments with a reported area. As indicated with the dashed orange line, the catchments with |*A*_rel_| above 50% was 8% (1,205 catchments). This analysis also shows that less than 17% of the catchments (2,712) had |*A*_rel_| above 10%.

Figure 4c focuses on catchments with |*A*_rel_| above 50% (1,205 catchments) and shows how the fraction of these catchment varies with catchment area. Notably, 17% of catchments under 100 km² exhibited |*A*_rel_| above 50%, while in all other ranges shown in the bar plot, the occurrence was below 5%. This analysis suggests that catchments with significant area differences tend to be relatively small.

Finally, for the 1,355 gauges (8% of the data) without catchment area information, the delineation was visually inspected, and a label was assigned to indicate the accuracy of the delineation based on the criteria shown in Table 3. Note that as it is not possible to calculate |*A*_rel_| for these catchments, the quality flags of “0” or “1” were never assigned to such basins. The visual inspection was again made using the river name, the river network provided by MERIT-Hydro and the EU-Hydro, Google Maps satellite imagery and nearby catchments delineated and labelled with a quality flag of “0”.

Hence, in the gauges’ layer stored in the final EStreams dataset, besides the original ***lat*** and ***lon*** coordinates, we included the ***lat_snap*** and ***lon_snap*** coordinates after the potential snap. The gauges layer also received an attribute called ***area_estreams***, which express the *A*_EStream_. Additionally, we included the *A*_rel_ as the attribute ***area_rel***, and the qualitative flag as the attribute ***area_flag***.

### Catchment aggregated data

The EStreams dataset includes streamflow, meteorological, and landscape variables. For streamflow, we distinguish between dynamic streamflow indices and hydro-climatic signatures, which are further detailed in their respective sections. Meteorological variables are discussed in the “Meteorological records” section. Finally, landscape attributes were categorized into six groups (Topography, Soils, Geology, Hydrology, Vegetation, and Land Cover) and are described in the “Landscape attributes” section. All catchment aggregations were derived using the catchment boundaries and areas calculated by EStreams. For example, all streamflow indices and signatures were computed using the specific discharge (in mm/day) derived with the *A*_EStreams_ areas.

#### Streamflow indices

In EStreams, streamflow data is presented in terms of “indices”, hence statistics of the daily data such as mean streamflow, maximum, minimum, percentiles and coefficient of variation, which are provided at annual, seasonal, monthly and weekly resolutions. The use of these indices is consistent with earlier works, such as the GSIM dataset^13,14^ and the CCl/WCRP/JCOMM Expert Team on Climate Change Detection and Indices (ETCCDI) (https://www.wcrp-climate.org/data-etccdi).

The use of indices instead of the daily data allows to make relevant climate information publicly available in cases where access to raw daily values is restricted. The selected indices, as discussed in the GSIM dataset^13,14^, are of high relevance and have been widely used in many hydrological studies, as they can facilitate the analysis of trends and changes in the regional water balance and the seasonal cycle.

The streamflow indices contained in EStreams are presented in Table 4, alongside with their units and temporal resolution. All the indices were computed for time-steps where at least 95% of the data was available, e.g., at annual time-step, the indices were computed for years where at least 347 days of data were available.

**Table 4:** Set of dynamic streamflow time series indices computed and made available at the present dataset.

Variable	Description	Units	Resolution
mean	Mean daily streamflow.	mm day^−1^	W, M, S and Y
std	Standard deviation of the daily streamflow.	mm day^−1^	W, M, S and Y
cv	Coefficient of the variation of the daily streamflow.	—	W, M, S and Y
min	Minimum daily streamflow.	mm day^−1^	W, M, S and Y
max	Maximum daily streamflow.	mm day^−1^	W, M, S and Y
min7	Minimum 7-day streamflow.	mm day^−1^	M, S and Y
max7	Maximum 7-day streamflow.	mm day^−1^	M, S and Y
p_{10, 20, 30, 40, 50, 60, 70, 80, 90}	Percentile values of the daily streamflow.	mm day^−1^	S and Y
iqr	Interquartile range of the daily streamflow (P75 minus P25)	mm day^−1^	W, M, S and Y
ct	Centre timing, which corresponds to the day of the year (doy) at which 50% of the annual flow is reached.	day	Y
doymin	The day of the year (doy) at which the minimum streamflow occurred.	day	Y
doymax	The day of the year (doy) at which the minimum streamflow occurred.	day	Y
doymin7	The day of the year (doy) at which the minimum 7-day streamflow occurred.	day	Y
doymax7	The day of the year (doy) at which the maximum 7-day streamflow occurred.	day	Y
gini	Gini coefficient	—	Y

#### Hydro-climatic signatures

In addition to the streamflow indices, we computed the same set of meteorological and hydrological signatures provided in the original CAMELS dataset^1^. Unlike streamflow indices, these signatures were calculated for the entire time period between 1950–2022 where data are available. Here we refer to these indices and signatures as hydro-climatic signatures (e.g., streamflow & precipitation mean, seasonality & aridity index, and runoff coefficient). For meteorology, we used precipitation and temperature derived from the Ensembles Observation (E-OBS) product^18^. This work used the “hydroanalysis” python package^38^ for the computation of these signatures.

The full list of signatures used is available in Table 5. We considered only catchments with more than one year of continuous measurements within the period of 1950–2022. Additionally, we also provide the number of years used for the signature’s computation (***num_years***), the start (***start_date***) and the end (***end_date***) of the observations between 1950–2022 to give a further overview of the period the signature refers to, considering separately the hydrological (***hydro***) and the climatic (***climatic***) signatures.

**Table 5:** Set of static hydro-climatic signatures.

Signature	Unit	Description
q_mean	mm day^−1^	Mean daily streamflow.
runoff_ratio	—	Ratio of mean daily streamflow to mean daily precipitation computed using Eq. (2) in Sawicz, 2011^100^.
q_elas_Sankarasubramanian	—	Streamflow precipitation elasticity. It represents the sensitivity of streamflow to changes in precipitation at the annual timescale computed using Eq. (7) in Sankarasubramanian, 2001^99^, the last element being P/Q not Q/P
slope_sawicz	—	Slope of the flow duration curve computed using Eq. (3) in Sawicz, 2011^100^.
baseflow_index		Ratio of mean daily baseflow to mean daily streamflow. Hydrograph separation performed using the Ladson, 2013^101^ digital filter.
hfd_mean	day of year	Mean half-flow date. It represents the date on which the cumulative streamflow reaches half of the annual discharge.
hfd_std	day of year	Standard deviation of the mean half-flow dates.
q_5	mm day^−1^	5% flow quantile, which represents low flows.
q_95	mm day^−1^	95% flow quantile, which represents high flows.
hq_freq	days yr^−1^	Frequency of Q > 9 times the median daily flow.
hq_dur	days	Average duration of flow events of consecutive days >9 times the median daily flow.
lq_freq	days yr^−1^	Frequency of Q < 0.2 times the median daily flow.
lq_dur	days	Average duration of flow events of consecutive days <0.2 times the median daily flow.
zero_q_freq	—	Frequency of days with Q = 0
p_mean	mm day^−1^	Mean daily precipitation.
pet_mean	mm day^−1^	Mean daily potential evapotranspiration (PET).
aridity	—	Ratio between PET and precipitation.
p_seasonality	—	Seasonality and timing of precipitation, which was estimated using the precipitation and temperature time series, and computed using Eq. (13) in Woods, 2009^102^.
frac_snow	—	Fraction of precipitation falling as on days colder than 0°C.
hp_freq	days yr^−1^	Frequency of P > 5 times the median daily precipitation (high precipitation events).
hp_dur	days	Average duration of periods with consecutive high precipitation events.
hp_time	season	Season during most high precipitation events occur (e.g., Fall, Winter, Summer or Spring).
lp_freq	days yr^−1^	Frequency of P events <1 mm day^−1^ (dry days).
lp_dur	days	Average duration of periods with consecutive dry days.
lp_time	season	Season during most dry days occur (e.g., Fall, Winter, Summer or Spring).
num_years_{hydro, climatic}	—	Number of years with hydrological or meteorological observations used for the signatures’ computation.
start_date_{hydro, climatic}	date	First date with with hydrological or meteorological observations used for the signatures’ computation.
end_date_{hydro, climatic}	date	Last date with hydrological or meteorological used for the signatures’ computation.

The hydrological year considered in this study starts at 1st of October and goes until the 30th of September. Unlike streamflow indices, these signatures are static, each represented by a single value calculated for the available data for the period from 1950 to 2022.

#### Meteorological records

EStreams used E-OBS^18^ for meteorological forcing data records, which has been widely used in hydrological studies over Europe^39–42^. E-OBS provides a pan-European observational dataset of surface climate variables that is derived by statistical interpolation of *in-situ* measurements, collected from national data providers. It is an open-access database with daily records ranging from 1950-present. We used the ensemble mean dataset at a resolution of 0.25 degrees. Additionally, we used the temperature records from E-OBS to derive potential evapotranspiration (PET) using the Hargreaves formulation^43^ and the “pyet” python package^44^ for computation. Each catchment has 9 daily meteorological time series associated with it, which are illustrated in Table 6. The accuracy of E-OBS may be dependent on station density^42^, which varies across Europe. In order to account for this potential source of uncertainty, EStreams also includes information on the number of weather stations and density aggregated to a buffer of 10 km within each catchment boundary.

**Table 6:** Meteorological catchment attributes at daily resolution from 1950 to 2022.

Group	Attribute	Description	Unit	Source
Meteorology	p_mean	Total mean daily precipitation measured as the height of the equivalent liquid water in a square meter.	mm day^−1^	E-OBS^18^
	t_{mean, min, max}	Daily mean, minimum and maximum air temperature measured near the surface.	°C	
	sp_mean	Mean air pressure at sea level.	hPa	
	rh_mean	Daily mean relative humidity measured near the surface.	%	
	ws_mean	Daily mean wind speed at 10-meter height.	ms^−1^	
	swr_mean	The flux of shortwave radiation (also known as solar radiation) measured at the Earth’s surface.	Wm^−2^	
	pet_mean	Potential evapotranspiration was estimated using the Hargreaves equation^43^.	mm day^−1^	derived
	stations_num_{p_mean, t_mean, t_min, t_max, sp_mean, rh_mean, ws_mean, swr_mean}	Number of weather stations measuring the given variable within the catchment boundary assuming a 10 km buffer.	-	E-OBS^18^
	stations_dens_{p_mean, t_mean, t_min, t_max, sp_mean, rh_mean, ws_mean, swr_mean}	Weather stations density for the given variable within the catchment boundary.	Stations km^−2^	

These attributes are aggregated over individual catchment boundaries. The table details both the time series variables and the information regarding the number of stations and their density.

#### Landscape attributes

A full overview of the landscape attributes contained in EStreams is shown in Table 7 and Table 8, with a short description, their units, and data provider. Regarding spatial coverage, except for the landcover & land use and soil types that have pan-European coverage, all the remaining products are global. Table 7 covers solely the fully static attributes, which are considered time invariant, such as elevation, soil types, main geology and mean vegetation indices. Conversely, Table 8 encompasses a group of attributes that are considered time variable, such as normalized difference vegetation index (NDVI), leaf-area index (LAI), irrigation and snow cover. These attributes are reported in time series at either monthly, yearly or in a specific number of years (e.g., irrigation and landcover) resolution.

**Table 7:** Set of static catchment attributes included in the present dataset.

Group	Attribute	Description	Unit	Source
Topography	ele_mt_{max, mean, min}	Mean, minimum and maximum elevation.	m	MERIT-Hydro^21,24^
	slp_dg_mean	Mean terrain slope.	°	
	flat_area_fra	Percentage of area with slope <3°.	%	
	steep_area_fra	Percentage of area with slope >15°.	%	
	elon_ratio	Derived elongation ratio^103^	—	
	strm_dens	Stream density, ratio of lengths of streams and the catchment area.	1000 Km km^-2^	
Soils*	root_dep	Depth available for roots.	cm	European Soil Database Derived data (ESDD)^28–30^
	soil_tawc	Total available water content.	mm	
	soil_fra_{sand, silt, clay, grav}	Sand, silt, clay and gravel fraction of soil material.	%	
	soil_bd	Bulk density.	g cm^−3^	
	oc_fra	Fraction of organic material.	%	
Geology	lit_fra_{class}	Percentage of each lithological class aggregated over the catchment.	%	Global Lithological Map Database (GLiM)^19^
	lit_dom	Lithological dominant class.	Classes (n = 16)	
	tot_area	Percentage of the catchment area covered by GLiM.	%	
	bedrk_dep	Depth to bedrock.	m	Pelletier, 2016^20^
Hydrology	dam_num	Number of dams upstream.	—	Georeferenced global Dams and Reservoirs^22^
	res_num	Number of reservoirs upstream.	—	
	dam_yr_{first, last}	First and last years of dam’s construction.	—	
	res_tot_sto	Total upstream storage volume.	10^6^ m^3^	
	lakes_num	Number of lakes upstream.	—	HydroLakes^45^
	lakes_tot_area	Total area covered by lakes upstream.	Km^2^	
	lakes_tot_vol	Total upstream volume.	10^6^m^3^	
Vegetation	ndvi_{month, mean}**	Mean NDVI over the catchment area.	—	MODIS^31^
	lai_{month, mean}**	Mean LAI over the catchment area.	—	MODIS^32^
Landcover	sno_cov_{month, mean}**	Mean snow cover percentage over the catchment area.	%	MODIS^27^

*All soil attributes were aggregated by mean, max, min, P05, P25, med, P75 and P90, which sums to a total of 64 variables.

**NDVI, LAI and snow cover attributes were aggregated considering the total mean and the month of the year (January = 01 to December = 12) mean from the period between 01.01.2001 to 31.12.2022, which means that each attribute has 13 variables here referred as static since not shown in a time series format.

**Table 8:** Set of the temporal catchment landscape attributes.

Group	Attribute	Description	Unit	Source
Vegetation	ndvi_mean	Monthly and yearly NDVI.	—	MODIS^31^
	lai_mean	Monthly and yearly LAI.	—	MODIS^32^
Landcover	sno_cov_mean	Monthly and yearly snow cover percentage time series.	%	MODIS^27^
	irrig_area_{yr}	10/5-year resolution total area equipped for irrigation.	km^2^	AEI_EARTHSTAT_IR product from HID^26^
	tot_area_{year}	Fraction of the catchment area covered by the Corine product.	—	CORINE^25^
	lulc_dom_{year}	Land cover majority class for 1990, 2000, 2006, 2012 and 2018.	Classes (n = 44)	
	lulc_{year}_{class}	Fraction of each landcover class aggregated over the catchment for 1990, 2000, 2006, 2012 and 2018.	—	

Vegetation and snow cover attributes have a monthly and yearly resolution from 2001–2022. The irrigation has a variable window resolution of 10–5-years from 1900–2005.

Topographical attributes were based on MERIT-Hydro^21^. Geology made use of the widely used Global Lithological Map Database (GLiM)^19^ and a gridded product for the estimation of the depth to bedrock^20^, which have been both used in several applications databases^1,8,23^. For the number of dams and of total upstream reservoir volume we used the Georeferenced global dams and reservoirs dataset^22^. A similar aggregation was performed for lakes using the HydroLakes dataset^45^. Vegetation indices and snow cover percentage made use of three MODIS products^27,31,32^ and were aggregated considering both temporal and static attributes. For irrigation, we decided to use the global dataset of the extent of irrigated land^26^, which ranges from 1900 to 2005, and has been already used in other studies^13,14,23^. The soil attributes were based on the European Soil Database Derived data (ESDD)^28,29,30^ and the land cover on the CORINE land cover dataset^25^. Both are widely used products which have been used in previous LSH datasets covering Europe^7,8^.

## Data Records

The current version of the EStreams dataset and catalogue (v1.0) is stored at a Zenodo repository^46^ at 10.5281/zenodo.13154470. The repository is organized into the following subfolders:

- ***streamflow_gauges:*** Contains two csv-files. One includes all the metadata associated with each of the 17,130 streamflow gauging stations such as location, river name, catchment area, and gauge elevation. The other file is the streamflow catalogue containing all the data provider information, further described in the following section.
- ***shapefiles:*** Contains two shapefiles. One shapefile includes the derived catchment boundaries associated with each streamflow gauge, and the other shapefile marks the location of the streamflow gauges. Both files are referenced in WGS 84.
- ***streamflow_indices:*** Contains one sub-folder per time resolution (weekly, monthly, seasonal and yearly) with a csv-file per computed index. The rows of each csv-file represent the time, and the columns represent the catchment.
- ***meteorology:*** Contains one csv-file per catchment (17,130 in total), each containing all the daily aggregated meteorological forcing records for that catchment (as detailed in Table 6). The rows of each csv-file represent the time, and the columns represent each of the 9 meteorological variables.
- ***attributes:*** Contains two sub folders. The ***static_attributes*** subfolder contains one csv-file per attribute group (i.e., topography, soils, geology, hydrology, vegetation and landcover) encompassing all the attributes shown in Table 7. The rows of the csv-file represent the gauging stations, and the columns represent the attribute variable. The ***temporal_attributes*** subfolder includes all the monthly or annual landscape attributes shown in Table 8. The csv-files in this subfolder are organized by gauging stations (rows), and attribute variables (columns), or as time series (each column represents one gauging station, and each row represents one date).
- ***hydroclimatic_signatures:*** Contains one csv-file with all computed hydro-climatic signatures for all catchments. The rows of each csv-file represent the streamflow gauging station, and the columns represent each of the 25 derived signatures.
- ***appendix:*** Contains three txt-files. One file provides descriptions of the lithological classes’ labels, another describes the landcover classes’ labels, and the third file includes licenses and data providers.

### Streamflow data catalogue

An important component of EStreams is the streamflow catalogue, which provides complete guidance on how to retrieve the raw streamflow data used in this study to compute the streamflow statistics. Table 9 provides an overview and description of the attribute fields included in the catalogue.

**Table 9:** Attribute fields included in the European Streamflow Catalogue provided.

Attribute name	Description
provider_id	Unique code used to refer the *basin_id* to their respective data provider
code_basins	Code shown in the first two-four digits of the *basin_id* of their respective catchments
provider_country	Country name of the data provided.
country_code	Country code of the data provided (e.g., PT for Portugal or AT for Austria).
provider_name	Name of the data provider.
license_redistribution	Type of redistribution license.
platform	Platform where the dataset is available. Either a website, or via contact request.
num_stations	Total number of streamflow stations available on the platform as of the date the catalogue data was derived.
start_date	Date of the first available streamflow measurement at the date of request/download.
end_date	Date of the last available streamflow measurement at the date of request/download.
website	Link to the official website of the data provider.
source_license	Link where the users can get further information regarding license and terms of use (when available).
source_streamflow	Link to the streamflow data provider website.
source_gauges_infos	Link to the official source where the gauges information is available (location, river and name).
references	Formal reference for citing the streamflow data.
observations	Extra information when needed to provide further guidance to the users.
download_method	Method of download available at the moment of publication. This specifies if users should download the data manually and individually, or if there is an official API, a provided code, or if a contact form is necessary to request the records.

Particularly, the field ***license_redistribution*** specifies the data redistribution policy of the data provider. In cases where this information is unavailable, users are advised to proceed with caution regarding any redistribution or specific use of the data, and to contact the data provider directly. The catalogue also includes various links to individual data providers, covering the website, the license source, streamflow and gauges metadata. Up to four different links are provided because the websites for downloading the streamflow time series may differ from those for the gauges metadata.

The Zenodo repository^46^ (10.5281/zenodo.13154470) supports versioning, which ensures reproducibility, benchmarking, and the extensibility of the dataset as new stations or time periods are added.

Additionally, Jupyter Notebook demonstrations are available at the GitHub repository^47^ (10.5281/zenodo.13255133) showing not only how to use the catalogue but also allowing to directly retrieve and pre-process each of the daily records currently included in EStreams. The repository is linked to a GitHub page, enabling users to track potential changes in data providers, websites, and propose updates. This collaborative approach can lead to new releases of the catalogue, ensuring EStreams remains an updated and dynamic resource.

### Gauges layer

A comprehensive overview of the gauges’ attributes and metadata included in this dataset is presented in Table 10. These attributes are designed to offer users complete guidance on data availability before downloading, thereby optimizing the data collection process. The attributes include the gauges names and location, data provider, topographic information, temporal data availability, quality and reliability descriptors, and nested catchments & flow order attributes. These attributes ensure that users have detailed information to facilitate the efficient retrieval and application of the streamflow data in various hydrological analyses.

**Table 10:** Description of the attributes of the streamflow gauges’ layer.

Attribute name	Description
basin_id	An 8-digit code defined by this work.
gauge_id	The official code available by the data source, which can be used to retrieve records directly from the data providers.
gauge_name	The official name of the station provided by the data source*.
gauge_country	Country code where the gauge is located (e.g., PT for Portugal or AT for Austria).
gauge_provider	Data source code aligned with the catalogue.
river	The name of the river provided by the data source*.
lon_snap	Longitude of the gauge in WGS84 original or moved.
lat_snap	Latitude of the gauge in WGS84 original or moved.
lon	Longitude of the gauge in WGS84 provided by the data source.
lat	Latitude of the gauge in WGS84 provided by the data source.
elevation	The official gauge elevation reported by the data provider*.
area_official	The official area reported by the data provider (*A*_official_)*.
area_estreams	The area (in km^2^) derived from the current delineation methodology (*A*_EStreams_).
area_flag	A quality flag for the current area computation as reported in Table 3.
area_rel	The percentual (%) relative difference between the derived and the reported area, relative to the reported area, as defined by Eq. (1).
start_date	First date with valid observations as of the date the data was accessed.
end_date	Last date with valid observations as of the date the data was accessed.
num_years	Number of years with valid data.
num_months	Number of months with valid data.
num_days	Number of days with valid data.
num_continuous_days	Maximum number of days between the *start_date* and *end_date* with no gaps.
num_days_gaps	Number of days with gaps between the *start_date* and *end_date*.
num_days_reliable	Number of days with data classified as “reliable” from the respective provider.
num_days_noflag	Number of days with data without a quality flag provided by the respective provider.
num_days_suspect	Number of days with data classified as “suspect” from the respective provider.
gauge_flag	Quality flag of the respective streamflow gauge as reported in Table 2.
duplicated_suspect	If it is the case, *basin_id* of the gauge suspect of being a duplicate with this gauge.
watershed_group	A number assigning to which main watershed is the gauge belongs to, e.g., all gauges within the Rhine watershed are assigned the number 1.
gauges_upstream	The number of unique gauging stations upstream of the given gauge. This count includes the basin itself but excludes any duplicate stations. This means that if one gauge has a duplicate, the count considers only one gauge.
nested_catchments	A list of all nested catchments within the given basin. This list includes the basin itself and may differ from the total number in *gauges_upstream* because it includes all gauges, retaining any duplicates within the same list.

*These are information seldom not available from official sources.

### Catchments layer

The delineated boundary of each catchment is stored in the catchment layer. This layer includes the ***basin_id*** field, which is also used for the gauges, allowing a link between the two datasets. Additionally, the catchment layer also has the fields ***gauge_id***, ***gauge_country*** (here named ***country***), ***area_official*** (here named ***area_offic***), ***area_estreams*** (here named ***area_estre***), ***area_flag***, ***area_rel***, ***start_date***, ***end_date***, ***gauge_flag***, ***gauges_upstream*** (here named ***upstream***) and ***watershed_group*** (here named ***group***), which were already described in Table 10. Note that ***area_official, area_estreams, gauge_country***, ***gauges_upstream*** and ***watershed_group*** had their names reduced due to storage limitations in the shape files. These fields ensure consistency between the catchment and gauge datasets, facilitating seamless integration and analysis.

## Technical Validation

### Duplicate stations

This work provides, alongside the gauges’ metadata, information on potential candidates for duplication. This information is useful for users aiming to have a consistent dataset for their hydrological analysis. The results indicate that a total of 885 gauges are identified as potential duplicates, representing about 5% of the total. This means that more than 16,600 gauges in the dataset may be seen as unique gauging stations. The duplicates are divided into two types: gauges duplicated with other gauges within the same provider and gauges duplicated with other gauges within different providers.

These first types of duplicates often occur when gauges are discontinued and later reactivated as new stations, usually resulting in stations with non-overlapping time records but located at the same point. These cases are primarily found in France (449) and Finland (160). For example, stations FR001479 (1969–1999), FR001477 (1993–1999) and FR001478 (2015–2023) are flagged as duplicate suspects among each other.

Additionally, 163 gauges are identified as duplicates across different data providers. These typically represent gauging stations located at the boundaries between countries and are mainly found in Austria (33), Switzerland (36) and Czech Republic (51). Interestingly, FR004543 is the only gauge identified as duplicate both within the same provider (FR002217) and across different providers (CH000268).

### Basin delineation validation

In this part of the study, we used the dataset provided by LamaH-CE^8^ for Austria, which includes both catchment boundaries and their respective officially reported areas. These were compared to the boundaries delineated using the methodology adopted in this work.

**Fig. 5:**
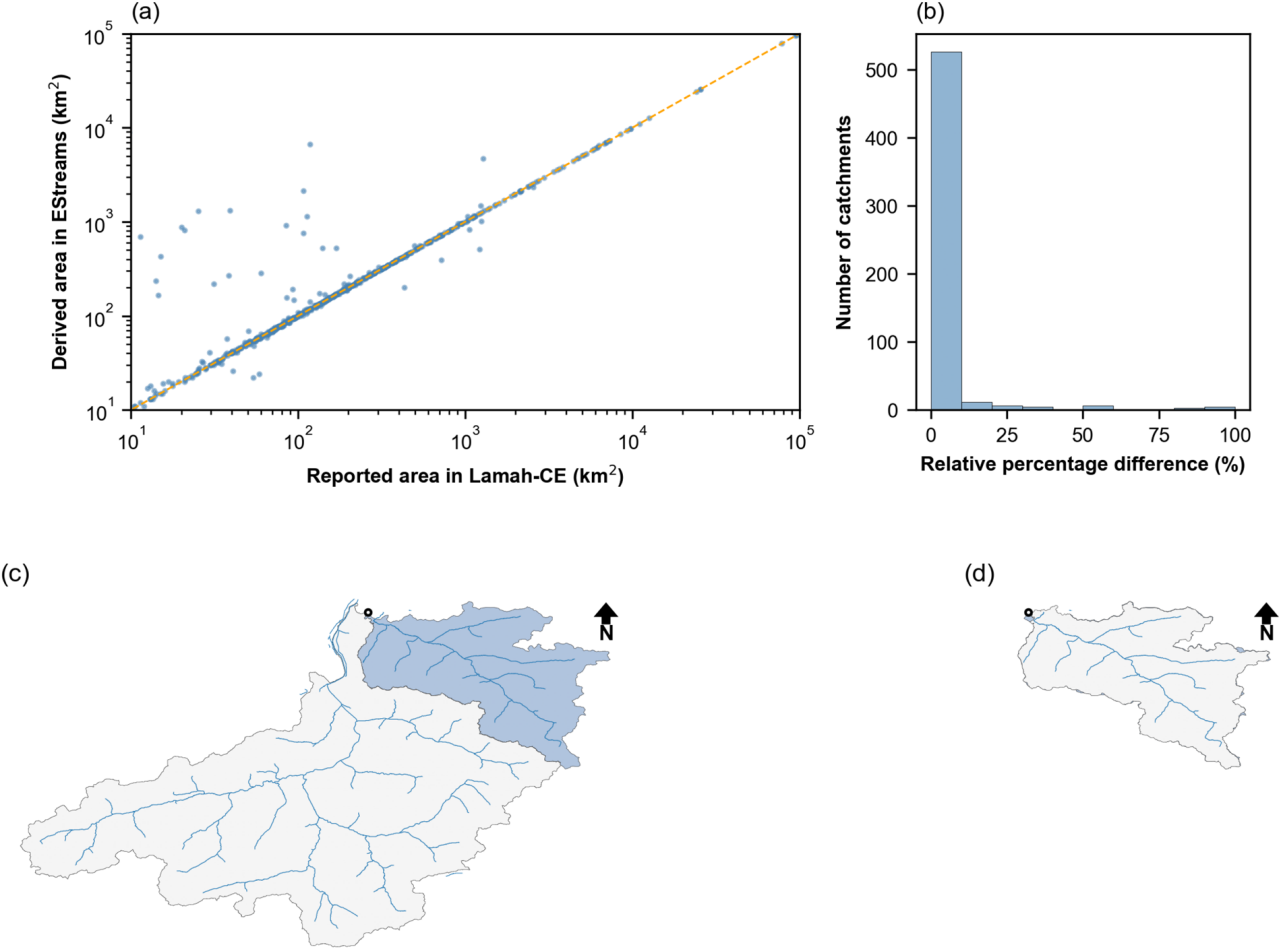
(**a**) Comparison of catchment boundary areas reported LamaH-CE^8^ against those delineated in this study. Both axes are presented in logarithmic scale to enhance visualization. (**b**) Histogram illustrating the |A_rel_| between the two sources of data. Most catchments exhibit |A_rel_| below 10%. Catchment AT000009 (EStreams) delineations are displayed (**c**) prior to manual adjustment of the outlet location and (**d**) following manual adjustment.

Figure 5a shows a scatter plot comparing the areas reported in LamaH-CE and those derived in EStreams. As expected, the scatter between the computed and reported areas is larger for smaller catchments. Figure 5b presents a histogram with the distribution of the relative absolute area difference |*A*_rel_| between the two areas (in %). Out of the total of 599 Austrian catchments, 539 had a |*A*_rel_| below 10%. This indicates that roughly 90% of the catchments were accurately delineated during the automatic part of the delineation process.

However, if we consider only catchments with areas above 100 km^2^ the number of catchments with |*A*_rel_| above 10% drops from 60 to only 21. After visual inspection, we concluded that the main cause of these discrepancies was associated either to the difficulties in the delineation of relatively small catchments, below 100 km^2^, or to small discrepancies between the streamflow gauge location in terms of the MERIT-Hydro network.

Figure 5c-d illustrate an example of the catchment delineation workflow for catchment AT000009. This catchment has an *A*_official_ of 1281.0 km^2^. Initially, *A*_EStream_ derived an area of 4680.0 km^2^, which accounts for a *A*_rel_ of +265.0%. Upon visual inspection, we realized that the inconsistency was due to the inaccurate location of the streamflow gauge in relation to the MERIT-Hydro River network (Fig. 5c). Since the outlet was not within the river network, the “delineator” python module used automatically moved it to the closest river network intersection, which had a much higher drainage area. After manually adjusting the streamflow gauge location, the delineation resulted in an area of 1,300.0 km^2^, an *A*_rel_ of only +1.5% (Fig. 5d).

### E-OBS assessment

#### Spatial coverage

EStreams used E-OBS to derive the catchment aggregated time series of meteorological variables. However, the number of stations used to produce the gridded dataset varies significantly from country to country. Here we provide a brief overview of the station densities used to derive the precipitation time series provided in E-OBS within each catchment. We present this analysis only for precipitation since it is considered the most important forcing input in hydrological studies and gives already a significant overview of the E-OBS network. To ensure a fair comparison, we considered a buffer of 10 km for the catchment boundaries and considered any station within this range to compute the number of stations.

**Fig. 6:**
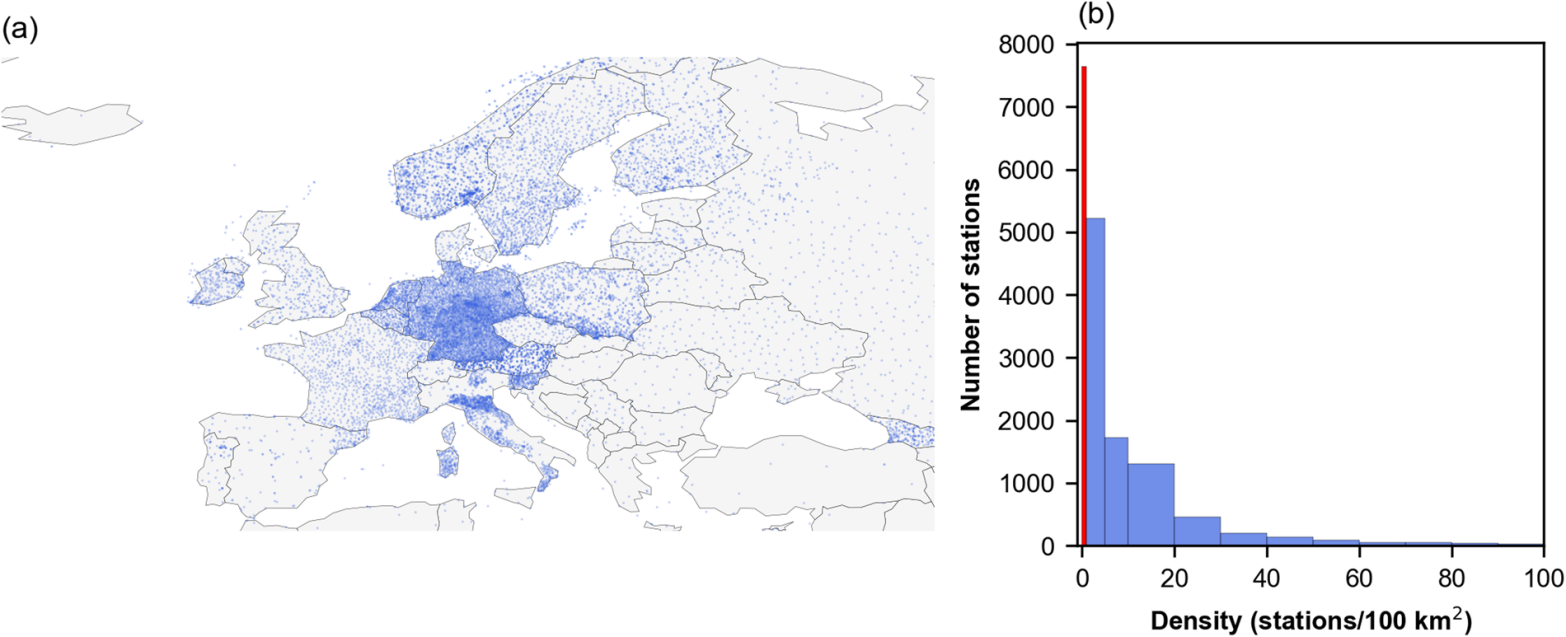
(**a**) Overview of the spatial distribution of the stations used to derive the precipitation time series grided data available at E-OBS^18^. (**b**) Histogram of the stations per catchment. Due to the high distribution of densities the bins are not evenly spaced, and the first bin (in red) corresponds to the threshold of one station per 100 km^2^. Basemap from GeoPandas^104^.

Figure 6a illustrates the spatial distribution of the stations, revealing a large spatial variability in station density. Central and North Europe exhibit the highest density, with Germany and Poland taking leading in station density, while the density decreases significantly towards South and East.

Figure 6b presents the histogram of the station density per catchment included in EStreams. The x-axis is resampled to stations per 100 km^2^ to facilitate visualization, with the threshold of less than one station per 100 km^2^ marked in red. A total of 9,840 catchments have at least one precipitation gauge per 100 km^2^. This represents, a median of 1.2 stations per 100 km^2^. Considering absolute terms, we found a total of 14,153 gauges with at least one precipitation station within their boundaries.

This information enables users to be aware of the highly variable quality of the provided E-OBS data and make informed decisions, especially considering the critical role of accurate precipitation data in many hydrological applications. Like streamflow data, national providers typically offer much higher resolution precipitation data compared to global databases^48^. While retrieving this information was beyond the scope of this study, users may choose to leverage such local data sources, particularly in regions where station density is notably low, such as in the South, East, and West of Europe.

#### Validation of meteorological forcing

We further validated the aggregated precipitation derived from E-OBS comparing it to the reported time series available at CAMELS-CH^7^ and CAMELS-GB^2^. Given that the aggregation of the forcing variables used E-OBS gridded data with a resolution of 0.25 degrees, we opted to include only catchments with areas above 100 km^2^ in the comparison.

**Fig. 7:**
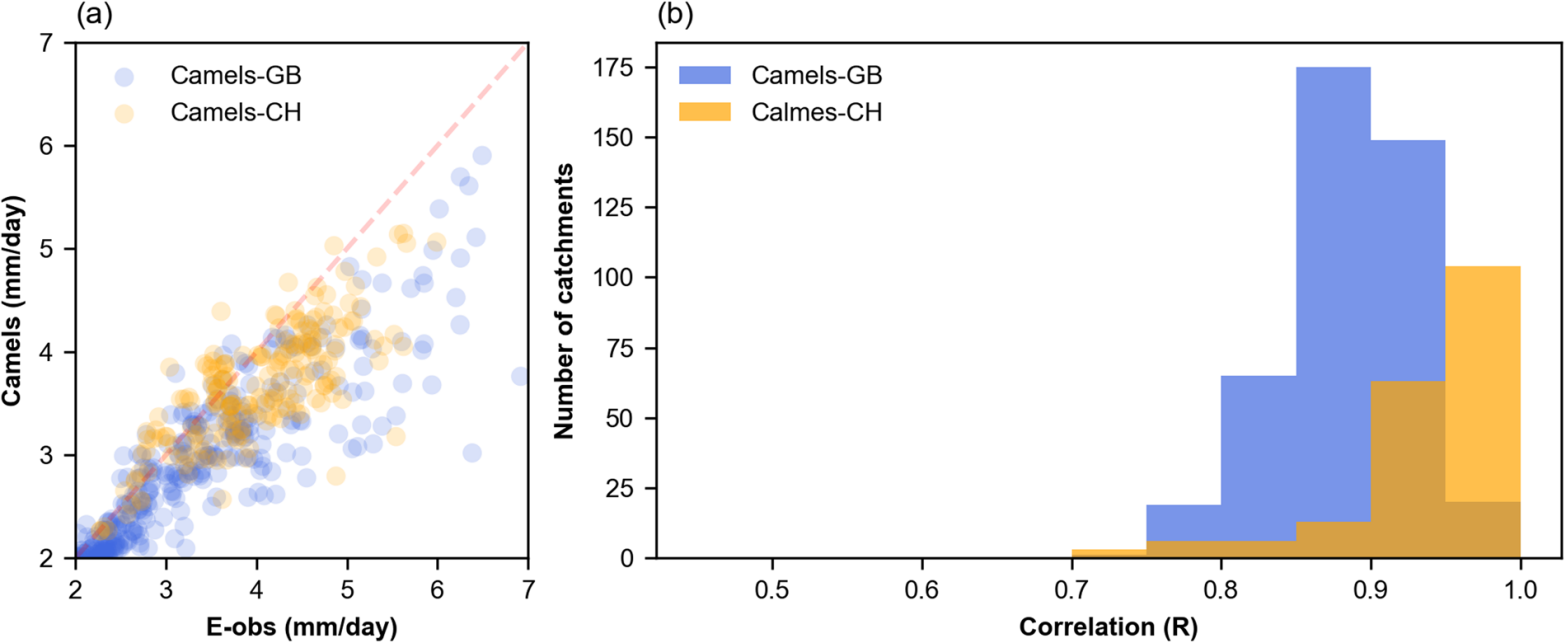
(**a**) Scatter plot of the long-term mean daily precipitation (1950–2022) considering the precipitation forcing time series derived from E-OBS^18^ and the provided in CAMELS-CH^7^ and CAMELS-GB^2^ and (**b**) Histogram of the correlation coefficient between the two data sources. The plots only show catchments with areas above 100 km^2^.

Figure 7a shows a scatter plot illustrating the daily precipitation from E-OBS and CAMELS. CAMELS-GB is represented in blue and CAMELS-CH in orange. A notable correspondence between the two sources is observable, with correlation coefficients of 0.89 for GB and 0.94 for CH. Generally, the scatter is lower in catchments with higher daily mean precipitation and an underestimation from E-OBS compared to the two sources is evident.

Figure 7b shows the distribution of the correlation coefficients between each daily time series of E-OBS and CAMELS. Again, it is possible to observe that most of the catchments presented a correlation above 0.8, indicating some agreement between the two precipitation sources. Overall, CAMELS-CH demonstrates higher correlation coefficients than CAMELS-GB. Despite this comparison only encompassing two different regions within the large span covered by EStreams, it was conducted using two independent sources. Hence, this analysis suggests that E-OBS, at least in countries where the station density is relatively high, provides a broadly consistent starting point for representing precipitation time series.

## Usage Notes

### Aggregated data

The original data used to aggregate the catchment attributes such as climate, geology, hydrology, land use and land cover, soil types and vegetation characteristics have all continental or global resolution. It should be kept in mind that such resolution is rather coarse compared to local information usually available at the national scales, but seldom easily accessible. We therefore recommend that users acknowledge these potential limitations when using the aggregated data. Additionally, we recommend users to also reference the original sources when using the aggregated data provided in EStreams.

### Streamflow catalogue

We recognize that potential retrospective check and updates of streamflow time series by the data providers may alter the information of the gauges metadata provided here. We also acknowledge that potential changes in the data providers’ platforms may alter the available links in the catalogue. Therefore, we invite the users to access the latest version of the catalogue and dataset on the Zenodo repository^46^ page for potential updates.

### Instructions for Python

We kindly request that future users of the EStreams’ codes read and follow carefully the instructions provided in the scripts. Specifically, (i) use the specified version of the Python modules (requirements.txt); (ii) clone the repository locally and keep all the original folders’ names; (iii) place the original data in their specified folder and with their expected filename and version; (iv) follow the pre-defined specified order of run for the available scripts (when necessary). Be aware that the potential main source of problems when running the scripts might be caused by not following these guidelines.

### Supplementary information

Supplementary Table 1

## Data Availability

The current version of the code used to produce the EStreams dataset and catalogue (v1.0.0) is available at a Zenodo repository^47^ at 10.5281/zenodo.13255133. For the latest version of the code, users are invited to visit the project GitHub repository at https://github.com/thiagovmdon/EStreams. The scripts are organized to enable users to follow a logical sequence during code usage. All data processing scripts are written in Python, while some data retrieval tasks are performed using JavaScript for the Google Earth Engine (GEE) platform. Although all scripts are executable, users must download and preprocess the original data due to redistribution licenses. Detailed instructions regarding the version used, data retrieval, and any required preprocessing are provided within the respective scripts.
