# Supplementary material for: EStreams: An integrated dataset and catalogue of streamflow, hydro-climatic and landscape variables for Europe
Source: Sci Data. 2024 Aug 13;11:879. doi: 10.1038/s41597-024-03706-1 (PMC11322309; doi:10.1038/s41597-024-03706-1)
Supplement: Supplementary file 1 — Supplementary Table 1 [file 41597_2024_3706_MOESM1_ESM.docx]

EStreams: An integrated dataset and catalogue of streamflow, hydro-climatic and landscape variables for Europe

**Authors**

Thiago V. M. do Nascimento^1,3^, Julia Rudlang^2^, Marvin Höge^1^, Ruud van der Ent^2^, Máté Chappon^4^, Jan Seibert^3^, Markus Hrachowitz^2^ and Fabrizio Fenicia^1^

**Affiliations**

1. Eawag: Swiss Federal Institute of Aquatic Science and Technology, Dübendorf, Switzerland

2. Department of Water Management, Faculty of Civil Engineering and Geosciences, Delft University of Technology, Delft, Netherlands

3. Department of Geography, University of Zurich, Zurich, Switzerland

4. Széchenyi István University, Department of Transport Infrastructure and Water Resources Engineering, Győr, Hungary

corresponding author: Thiago Nascimento (thiago.nascimento@eawag.ch)

# Supplementary material

| Data provider | Original quality flag | Reliable | Suspect | No-flag | Missing |
| --- | --- | --- | --- | --- | --- |
| FRANCE | 12: Doubtful  16: Unqualified  20: Good | 20 | 12 | 16 | - |
| PORTUGAL | (!): to be updated: (vau): Automatic measurer.  (vco): Conventional measurer.  (ei): Value provided by INAG.  (vdd): Different values from (vco) and (vau). | (vau), (vco), (ei) | (!), (vdd) | - | - |
| IRELAND (EPA) | Suspect, Fair,  Good, Poor, Excellent, Estimated, Unchecked, Extrapolated | Fair, Good, Excelent | Suspect, Poor, Estimated, Extrapolated | Unchecked | - |
| IRELAND (OPW) | 31: Good quality estimated data and validated with good quality level.  32: Good quality estimated data and validated with corrected water level.  36: Data may contain a fair degree of error.  46: Data may contain a significant degree of error.  56: Extrapolated data.  96: Provisional data.  101: Estimated using an unreliable water level.  151: unusable data.  254: Provisional data.  255: Missing data. | 31, 32, 36 | 46, 56, 96, 101, 151, 254 | 255 |  |
| ITALY (ISPRA) | 1: Validated  NaN: Missing | 1 | NaN | - | - |
| ITALY (TOSCANA) | R: Reconstructed  V: Validated  @: Missing  P: Pre-validated | V | R, P | - | @: |
| ITALY (LOMBARDIA) | 1: Good quality recorded data  2: Good quality processed data  30: Irregular sampling,  60: Out of range  70: Estimated data  77: Data from correlation with another gauging station  82: Data from linear interpolation  130: Rough estimation  140: Data to be checked  149: Measurement below detection limit  151: Missing data  160: Out of range  201: Missing data  202: Suspect  255: Suspect | 1, 2, 30, 77, 82, | 60, 70, 130, 149, 160 | 140 | 151 |
| ITALY (LIGURIA) | 1: Validated data  4: Non-validated data | 1 | 4 | - | - |
| SWEDEN | Kontrollerat: Controlled data  Okontrollerat: Not controlled data  Grovt kontrollerat: Roughly controlled data | Kontrollerat, Grovt kontrollerat | Okontrollerat | - | - |
| DENMARK | GODK: OK  UK: UNKNOWN | GODK | - | UK | - |

**Supplementary Table 1.** Overview of the original quality flag for each of the countries which provide them, and their respective translation to our work.
